# Functional Outcomes and Complications of Non-surgical Management for Midshaft Clavicle Fractures

**DOI:** 10.7759/cureus.95788

**Published:** 2025-10-31

**Authors:** Tariq Ahmad, Khalid Khan, Kamran Khan, Ibrahim Khan, Muhammad Tayyab, Abdus Samad Khan

**Affiliations:** 1 Department of Orthopedic Surgery, MTI Mardan Medical Complex, Mardan, PAK; 2 Department of Orthopedics and Trauma, MTI Mardan Medical Complex, Mardan, PAK; 3 Department of Orthopedics, MTI Mardan Medical Complex, Mardan, PAK; 4 Department of Trauma and Orthopedics, Bradford Teaching Hospitals NHS Foundation Trust, Bradford, GBR

**Keywords:** clavicle fractures, conservative treatment, disabilities of the arm, fracture, fracture healing, malunited fractures, nonunion, shoulder and hand score, treatment outcome

## Abstract

Introduction

Midshaft clavicle fractures are commonly managed conservatively, especially in resource-limited settings. While union rates are generally high, concerns remain regarding functional outcomes and complications such as nonunion and malunion.

Objective

This study’s objective is to evaluate the long-term functional outcomes and complications associated with non-surgical management of middle-third clavicular fractures.

Methodology

This prospective observational study was conducted at Mardan Medical Complex over 24 months. A total of 110 patients with isolated, closed, middle-third clavicle fractures treated conservatively were enrolled. Functional outcomes were assessed using the QuickDisabilities of the Arm, Shoulder, and Hand (QuickDASH) score and Constant-Murley Shoulder Score (CMSS) at 12-month follow-up. Radiological union, complications, and patient satisfaction were also recorded. Statistical analysis was performed using SPSS version 25.0 (IBM Corp., Armonk, NY). Independent t-tests and chi-square tests were applied where appropriate, with a p-value of < 0.05 considered statistically significant.

Results

Out of 110 enrolled patients, 102 (92.7%) completed follow-up. Union was achieved in 88 (86.3%) patients within 16 weeks. Nonunion occurred in seven (6.9%), and malunion in 10 (9.8%). The mean DASH score was 9.6 ± 7.2, and CMSS was 88.2 ± 6.8. Patients with union had significantly better scores (p < 0.001). Cosmetic dissatisfaction was reported in 18 (17.6%) patients, mostly associated with malunion.

Conclusion

Conservative management of midshaft clavicle fractures is generally effective, but functional outcomes are significantly influenced by healing quality. Careful patient selection and follow-up are essential to minimize complications and improve outcomes.

## Introduction

Clavicle fractures are among the most common skeletal injuries encountered in clinical practice, accounting for approximately 2.6-3% of all fractures and up to 44% of injuries involving the shoulder girdle [1]. The majority of clavicle fractures (~80%) occur in the middle third of the bone due to its anatomical vulnerability  [2]. These injuries are particularly prevalent among young, active individuals and often result from direct trauma, such as road traffic accidents, sports injuries, or falls  [3]. Given the superficial location and biomechanical role of the clavicle in connecting the axial skeleton to the upper limb, fractures in this region can lead to significant functional limitations and discomfort if not managed properly [4]. Traditionally, midshaft clavicular fractures have been treated non‑surgically using slings, figure-of-eight bandages, or simple arm supports. This approach has been favored due to generally high union rates, low complication risks, and the avoidance of surgical complications such as infection, neurovascular injury, or implant failure  [5]. Numerous earlier studies have reported satisfactory functional and cosmetic outcomes with conservative management, particularly in non-displaced or minimally displaced fractures [6].

However, over the past two decades, scrutiny over long-term non-surgical outcomes has increased. Emerging studies have highlighted issues including higher rates of symptomatic malunion, nonunion, persistent shoulder dysfunction, and dissatisfaction with cosmetic results [7]. Functional impairments, such as reduced shoulder strength, range of motion, and endurance, may significantly affect quality of life, especially in young, active patients [8]. Some data suggest that fractures initially deemed stable may still develop complications over time, possibly due to inadequate immobilization or patient non-compliance  [9].

Despite growing literature comparing surgical and non-surgical approaches, most studies have focused on short- to medium-term outcomes. Long-term functional recovery and patient-reported outcomes in conservatively managed patients remain under-reported [10]. Furthermore, variability in fracture characteristics, patient demographics, rehabilitation protocols, and outcome measures complicates the establishment of standardized guidelines [11]. Many published studies originate from high-resource settings, limiting their generalizability to regions where surgical care is less accessible or conservative management is the primary treatment option.

There is limited local evidence assessing the long-term functional outcomes and complications of non-surgical management for middle third clavicular fractures, particularly in low-resource settings; this study aims to evaluate these outcomes to inform evidence-based clinical decision-making.

## Materials and methods

Study design and setting

In this study, “conservative management” refers to non-operative treatment, which included immobilization using either a simple arm sling or a figure-of-eight clavicle brace. Both methods aimed to maintain shoulder immobilization and comfort during fracture healing. The selection between the two devices was based on patient comfort and clinician preference; however, all patients followed a standardized rehabilitation protocol consisting of gradual passive and active shoulder exercises after immobilization removal. This standardization minimized treatment bias across subgroups.

Sample size calculation

Recent clinical studies have reported a complication rate, specifically nonunion or malunion, of approximately 15-20% in conservatively treated midshaft clavicle fractures, particularly in displaced cases [12]. Based on these data, a conservative estimated proportion (p) of 0.18 was used to calculate the required sample size using the standard formula for a single proportion [13,14]:

$n =\frac{Z^{2}\text{ x }p \text{x} (1-p)}{d^{2}}$

Where Z = 1.96 (for a 95% confidence level), p = 0.18 (anticipated complication rate), and d = 0.08 (margin of error).

After adjusting for an anticipated 20% loss to follow-up over the 12-month evaluation period, the final required sample size was 110 participants. This sample size was sufficient to ensure statistical power and precision for detecting clinically meaningful differences in outcome measures, while remaining feasible within the study duration and institutional patient flow.

Inclusion and exclusion criteria

Individuals who had suffered an isolated, closed, non-pathological fracture of the middle third of the clavicle and were between 18 and 60 years of age were eligible to participate in the study. Participants who presented within 10 days of injury and were treated non-surgically using either an arm sling or a figure-of-eight brace were included. All patients provided written informed consent and agreed to a minimum follow-up period of one year.

Exclusion criteria included polytrauma patients, open fractures, associated neurovascular injuries, and those with a history of pre-existing clavicular or shoulder pathology. In addition, patients who were lost to follow-up before the 12-month evaluation period were excluded from the final analysis.

Patient enrollment and treatment protocol

A total of 110 patients were consecutively enrolled through the orthopedic outpatient department and emergency services. Once radiological confirmation of a midshaft clavicular fracture was established using a standard anteroposterior (AP) and 15° cephalic tilt view, all patients were managed conservatively, either with an arm sling or a figure-of-eight brace, depending on patient comfort and surgeon preference.

During the first two to three weeks, patients were advised to restrict shoulder motion to facilitate early healing. Afterward, a graduated rehabilitation program was initiated, beginning with pendulum and passive range-of-motion exercises, followed by active movement and strengthening exercises once radiological evidence of union appeared. This protocol ensured consistency in non-surgical management and minimized functional bias between treatment approaches.

Follow-up and outcome assessment

Patients were reviewed at two weeks, six weeks, three months, six months, and 12 months post-injury. At each visit, a detailed clinical assessment was performed, including evaluation of pain, shoulder function, range of motion, and evidence of fracture healing or complications. At the two-week follow-up, only passive range of motion (such as pendulum exercises) was assessed to avoid stress on the healing fracture. Full active range-of-motion testing commenced after six weeks, once early radiological union was confirmed. Radiographs obtained at six weeks, three months, and 12 months were used to determine radiological union. The follow-up schedule and assessment methods were consistent with established orthopedic protocols [15-19].

The primary outcome measure was functional recovery at 12 months, assessed using the QuickDASH (Quick Disabilities of the Arm, Shoulder, and Hand) score [15-17] and the Constant-Murley Shoulder Score (CMSS) [18,19]. The QuickDASH, a validated short-form version of the original DASH questionnaire [20], includes 11 items covering pain, symptoms, and upper limb function. It has demonstrated high correlation (r > 0.9) with the full DASH and offers excellent reliability, validity, and patient compliance during serial assessments. Secondary outcomes included the rates of non-union, malunion, delayed union, cosmetic dissatisfaction, shoulder strength, and range-of-motion limitations. Malunion was defined as >15° angulation or ≥1.5 cm shortening on radiographs, while delayed union was defined as the absence of bridging callus at 16 weeks. Nonunion was defined as the absence of bone healing beyond 24 weeks. Patients with delayed or nonunion were managed with continued functional support and physiotherapy; surgical conversion was considered only in persistent nonunion cases.

Data analysis

All data were entered and analyzed using SPSS version 25 (IBM Corp., Armonk, NY). Descriptive statistics were applied to summarize demographic characteristics, clinical parameters, and outcome measures. Continuous variables such as age, healing time, QuickDASH scores, and CMSSs were expressed as mean ± standard deviation (SD), while categorical variables such as gender, fracture laterality, and occurrence of non-union or malunion were presented as frequencies and percentages.

Comparisons of pre- and post-treatment functional scores were performed using the paired t-test or Wilcoxon signed-rank test, depending on data normality. The chi-square test (or Fisher’s exact test when appropriate) was used to evaluate associations between categorical variables such as fracture pattern, management type, and outcome.

All statistical tests were two-tailed, and a p-value < 0.05 was considered statistically significant. The analytical approach ensured robust evaluation of the relationship between treatment modality and mid-term functional outcomes while accounting for both parametric and non-parametric data distributions.

## Results

Out of 110 patients initially enrolled, 102 (92.7%) patients completed the 12-month follow-up, while eight (7.3%) patients were lost. The mean age was 34.5 ± 10.2 years, ranging from 18 to 58 years. The majority were male (n = 72, 70.6%), while females comprised 30 (29.4%) of the study population. Injuries affected the right clavicle in 64 (62.7%) patients and the left side in 38 (37.3%). Most participants were treated with a simple arm sling (68, 66.7%), while 34 (33.3%) patients used a figure-of-eight brace. The average time from injury to initiation of treatment was 3.4 ± 1.8 days. Table 1 provides a summary of these baseline characteristics.

**Table 1:** Baseline demographics of participants (n = 102). “Figure-of-eight” refers to a figure-of-eight clavicle brace used for conservative management. n = number of patients; SD = standard deviation

Variables	Mean ± SD or number (%)
Age (years)	34.5 ± 10.2
Gender	-
Male	72 (70.6%)
Female	30 (29.4%)
Side of injury	-
Right	64 (62.7%)
Left	38 (37.3%)
Treatment type	-
Arm sling	68 (66.7%)
Figure-of-eight brace	34 (33.3%)
Time from injury to treatment (days)	3.4 ± 1.8
Comorbidities and risk factors	-
Smokers	18 (17.6%)
Chronic steroid or OCP use	6 (5.9%)
Autoimmune/biologic therapy	3 (2.9%)
Diabetes mellitus or hypertension	12 (11.8%)

**Figure 1:**
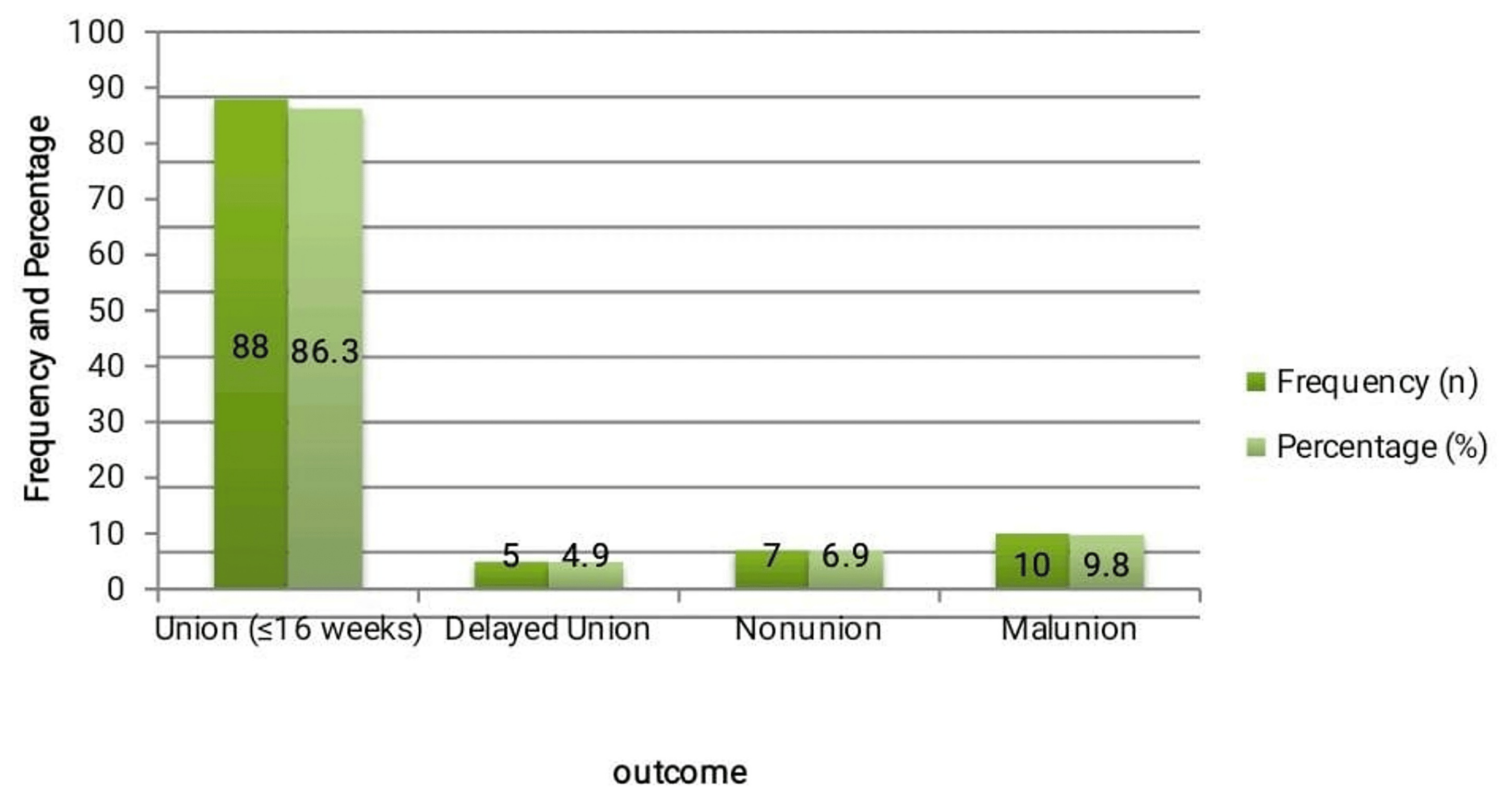
Distribution of fracture-healing outcomes following conservative management (n = 102). Bar graph illustrating the frequencies and percentages of patients achieving union, delayed union, nonunion, and malunion after 12 months of follow-up. Radiological union was defined as cortical continuity on AP and axial clavicle views; delayed union > 16 weeks, nonunion > 24 weeks, and malunion as healed fracture with > 15° angulation or ≥ 1.5 cm shortening.

Out of 102 patients, 88 (86.3%) achieved complete radiological union within 16 weeks of conservative management. Delayed union was observed in five (4.9%) patients, whereas nonunion occurred in seven (6.9%). Malunion was noted in 10 (9.8%) cases, most of which were associated with marked initial displacement. The mean time to fracture union was 12.4 ± 3.6 weeks, with the majority achieving healing between 10 and 14 weeks post-injury (Figure 1). These findings demonstrate a high overall success rate of nonoperative management, although notable complications were observed in approximately one out of every seven patients.

**Figure 2:**
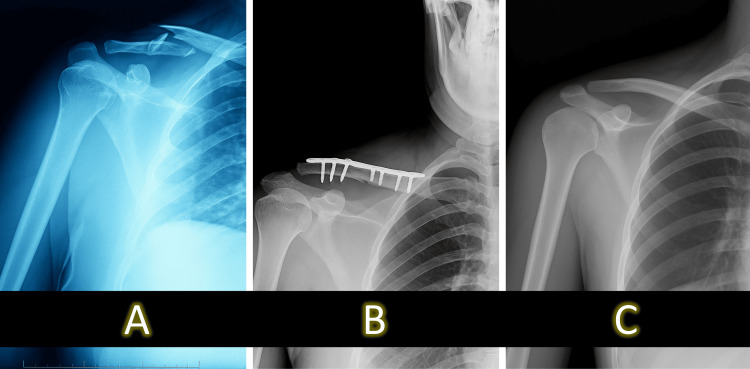
A representative case sequence illustrating the natural course of a displaced midshaft clavicle fracture. (A) Initial radiograph showing a completely displaced midshaft clavicular fracture with superior displacement of the medial fragment. (B) Postoperative radiograph following anatomical reduction and fixation using a contoured plate and screws. (C) Follow-up radiograph at 24 weeks demonstrating complete cortical union and restored alignment.

**Figure 3:**
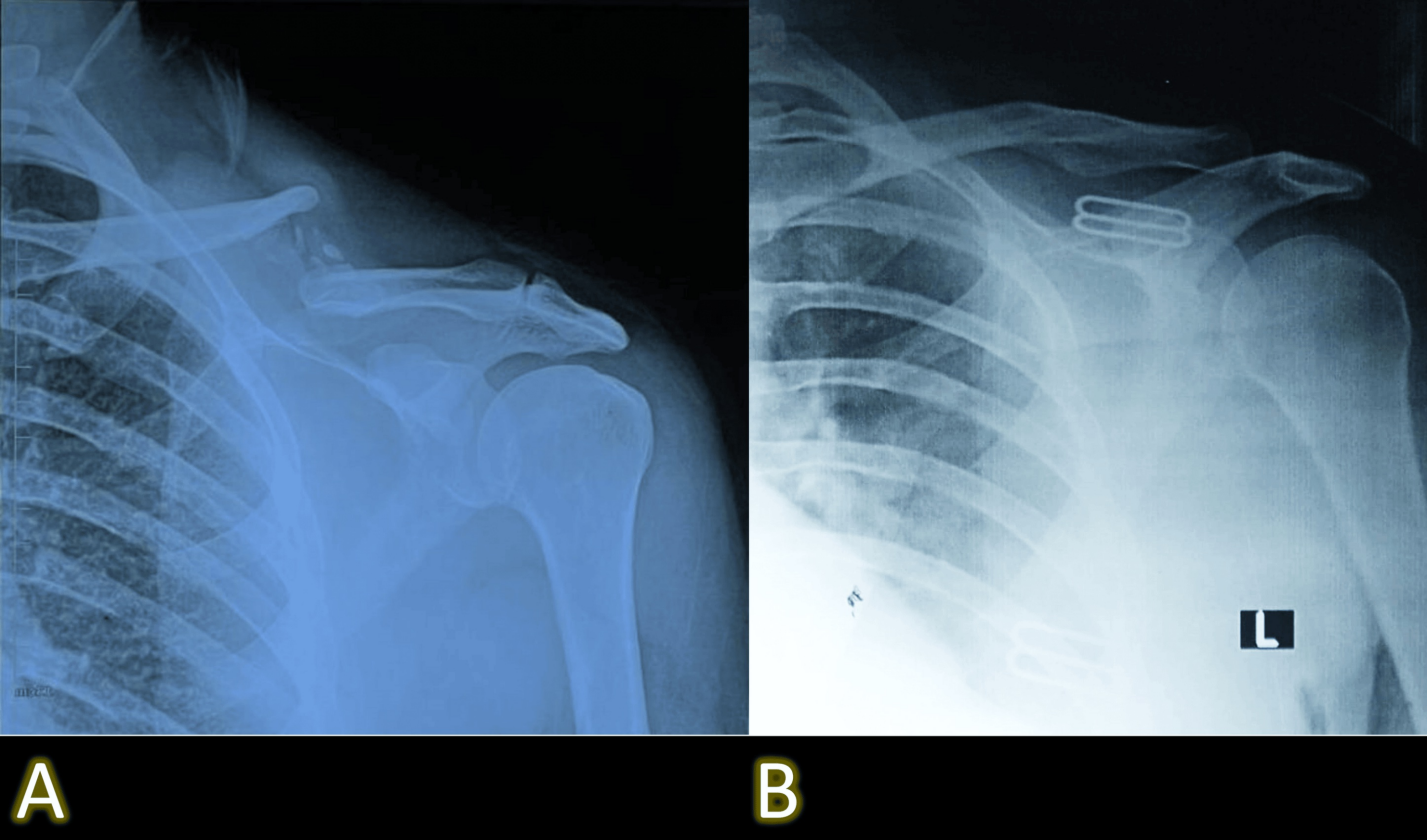
Radiographic comparison demonstrating nonunion and eventual healing following conservative management. (A) Nonunion evident at 24-week follow-up. (B) Healed fracture at 52-week follow-up.

Among patients with delayed union (n = 5), continued conservative care with extended immobilization followed by structured physiotherapy resulted in complete healing by approximately 22 weeks. Of the seven patients who developed nonunion, four underwent surgical fixation with open reduction and internal plating after six months due to persistent pain and radiographic non-healing, while three declined surgery and were managed functionally through activity modification and supervised physiotherapy. Representative radiographs of typical fracture progressions and delayed healing are shown in Figure 2 and Figure 3.

The mean DASH score for all patients at final follow-up was 9.6 ± 7.2, indicating minimal overall disability. Patients who achieved union demonstrated significantly better functional outcomes, with a mean DASH score of 7.8 ± 5.6, compared to 19.6 ± 6.7 in those with nonunion or malunion. Similarly, the mean CMSS was significantly higher in the union group (90.5 ± 5.2) than in the complication group (75.2 ± 6.1). These differences were statistically significant (p < 0.001) for both scores, as assessed using the independent t-test. These results highlight the strong positive association between radiological union and improved functional performance at one year post-injury. The summary of these outcomes is presented in Table 2.

**Table 2:** Functional scores at final follow-up (n = 102). Functional outcomes were assessed using the Quick Disabilities of the Arm, Shoulder, and Hand (DASH) score and the Constant-Murley Shoulder Score (CMSS). An independent t-test was used to compare mean scores between the union and nonunion/malunion groups. A p-value < 0.05 was considered statistically significant.

Group	DASH score (mean ± SD)	CMSS (mean ± SD)
All patients	9.6 ± 7.2	88.2 ± 6.8
Union group (n = 88)	7.8 ± 5.6	90.5 ± 5.2
Nonunion/malunion (n = 17)	19.6 ± 6.7	75.2 ± 6.1
p-value	< 0.001	< 0.001

**Figure 4:**
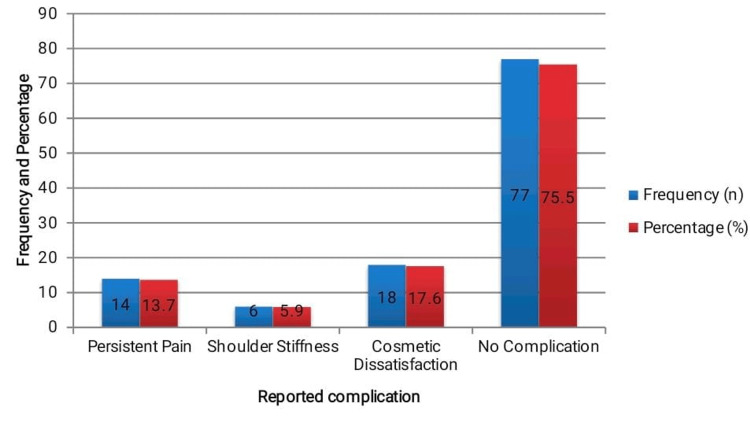
Reported complications and satisfaction (n = 102). Frequencies and percentages represent reported complications and satisfaction at 12-month follow-up. A chi-square test was used to assess the association between malunion and cosmetic dissatisfaction. A p-value < 0.05 was considered statistically significant.

A total of 25 (24.5%) patients experienced at least one complication. The most frequently reported issue was persistent pain in 14 (13.7%) patients, followed by cosmetic dissatisfaction in 18 (17.6%) patients and shoulder stiffness in 6 (5.9%) patients. Among those with cosmetic complaints, 13 (72.2%) had radiographic evidence of malunion. A chi-square test revealed a statistically significant association between malunion and cosmetic dissatisfaction (χ² = 9.53, p = 0.002), indicating that altered bone alignment may impact patient-perceived aesthetics. The majority of patients without complications (77, 75.5%) reported high satisfaction with the outcome. The breakdown is illustrated in Figure 4.

When analyzing the relationship between fracture healing and functional recovery, a strong statistical difference was observed. Patients who achieved full union (n = 88) demonstrated significantly better functional scores, with a mean DASH score of 7.8 ± 5.6 and a CMSS of 90.5 ± 5.2. In contrast, patients with nonunion or malunion (n = 17) had a substantially higher DASH score of 19.6 ± 6.7, indicating more disability, and a lower CMSS of 75.2 ± 6.1, reflecting poorer shoulder function. These differences were analyzed using the independent t-test, which revealed statistically significant results for both scoring systems (t = 6.42 for DASH and t = 7.01 for CMSS, p < 0.001 for both). These findings strongly support the conclusion that optimal fracture union is directly associated with improved long-term functional outcomes, as detailed in Table 3.

**Table 3:** Association of healing type with functional outcomes (n = 105). Independent t-test was used to compare functional scores between healing types. Significant differences were observed in both Disabilities of the Arm, Shoulder, and Hand (DASH) score and the Constant-Murley Shoulder Score (CMSS) scores (p < 0.001).

Healing type	n	DASH Score (mean ± SD)	CMSS (mean ± SD)	t-value	p-value
Union	88	7.8 ± 5.6	90.5 ± 5.2	6.42	<0.001
Nonunion/malunion	17	19.6 ± 6.7	75.2 ± 6.1	7.01	<0.001

## Discussion

This study evaluated the functional outcomes and complications associated with non-surgical management of middle-third clavicle fractures at a 12-month follow-up. A high rate of successful union was observed, with 86.3% of patients achieving radiological healing within 16 weeks. Functional outcomes measured through QuickDASH and Constant-Murley scores were generally favorable among patients who achieved complete union. However, complications such as malunion, nonunion, and cosmetic dissatisfaction were notable and significantly influenced patient-reported outcomes. Although the present study provides valuable insights into short- to mid-term recovery, it is acknowledged that true long-term functional outcomes-particularly those reflecting late-onset symptoms, activity resumption, and shoulder endurance-are best evaluated over a period of three to five years. Therefore, extended follow-up studies are warranted to better characterize the durability and long-term implications of conservative management in this patient population.

When compared to existing literature, the findings align with prior studies reporting union rates of approximately 80-85% with conservative management of midshaft clavicle fractures [21]. Similar studies have documented nonunion rates ranging from 13-20%, depending on patient selection and displacement characteristics [22,23]. The malunion rate observed in the current study is consistent with prior data, suggesting that displacement increases the likelihood of healing in a shortened or angulated position [24]. Moreover, the observed cosmetic dissatisfaction in 17.6% of patients parallels reports where over 15% of conservatively managed individuals expressed concern about visible deformity or asymmetry, especially in cases of malunion [25]. Functional outcome scores in this study, including a mean DASH of 9.6 and CMSS of 88.2, fall within the range reported in prior literature for similar cohorts, particularly in studies with 12-month or longer follow-ups [26]. However, studies favoring surgical fixation often report slightly better scores and lower complication rates, especially in active individuals and those with completely displaced fractures [27]. Despite overall good outcomes, this study emphasizes that non-surgical treatment is not without its drawbacks. The association between malunion and both functional impairment and cosmetic dissatisfaction highlights the need for careful patient selection. Displaced fractures, in particular, may benefit from closer monitoring or early surgical consultation if poor alignment is suspected [28].

Systematic reviews and large cohort studies comparing the surgical and non-operative treatment of midshaft clavicle fractures provide additional support for these findings. While non-operative management results in acceptable functional outcomes for most patients, operative treatment tends to reduce the risk of nonunion and achieve better anatomical alignment [29]. Randomized controlled trials and meta-analyses consistently show that operative fixation yields significantly lower nonunion and symptomatic malunion rates and provides superior short-term functional outcomes, particularly in cases of displaced fractures [30]. Long-term follow-up studies of conservatively treated fractures indicate that greater displacement and shortening are associated with increased symptoms, worse ASES and SPADI scores, and more lasting functional impairment even after 10-30 years [31]. However, several studies note that when nonunion and malunion cases are excluded, late functional outcomes between operative and non-operative groups may converge, with DASH and Constant scores tending to be comparable at one year or later [32].

Limitations and future suggestions

This study has various limitations. Firstly, the results' generalizability may be impacted by the fact that it was carried out at a single location with a small sample size. Second, radiological assessment did not include three-dimensional imaging, which may have underestimated subtle malalignment. Third, the study did not directly compare surgical and non-surgical groups, which would have provided a more robust understanding of relative outcomes. Finally, outcomes were measured at 12 months; longer follow-up may reveal late-onset complications or functional decline.

Future studies should involve multicenter designs with larger sample sizes and stratify patients based on displacement and activity level. Randomized controlled trials comparing surgical versus conservative treatment would help clarify the indications for operative intervention. Moreover, incorporating patient-reported outcome measures focused on aesthetics and quality of life could provide deeper insight into the psychosocial impacts of clavicular deformity following non-surgical treatment.

## Conclusions

Middle-third clavicle fractures managed non-surgically generally demonstrate a high rate of fracture union and favorable long-term functional outcomes. Nevertheless, a substantial subset of patients experience complications such as malunion, nonunion, and cosmetic dissatisfaction, which were significantly associated with lower functional scores in this cohort. These findings underscore the importance of careful patient selection, individualized treatment planning, and close radiological follow-up, particularly in fractures with notable displacement. Although most patients can achieve satisfactory recovery with conservative management, adopting a personalized approach to treatment decisions remains crucial for optimizing both clinical and functional outcomes.
